# Supplementary figures and images for: Treponema denticola dentilisin triggered TLR2/MyD88 activation upregulates a tissue destructive program involving MMPs via Sp1 in human oral cells
Source: PLoS Pathog. 2021 Jul 13;17(7):e1009311. doi: 10.1371/journal.ppat.1009311 (PMC8301614; doi:10.1371/journal.ppat.1009311)

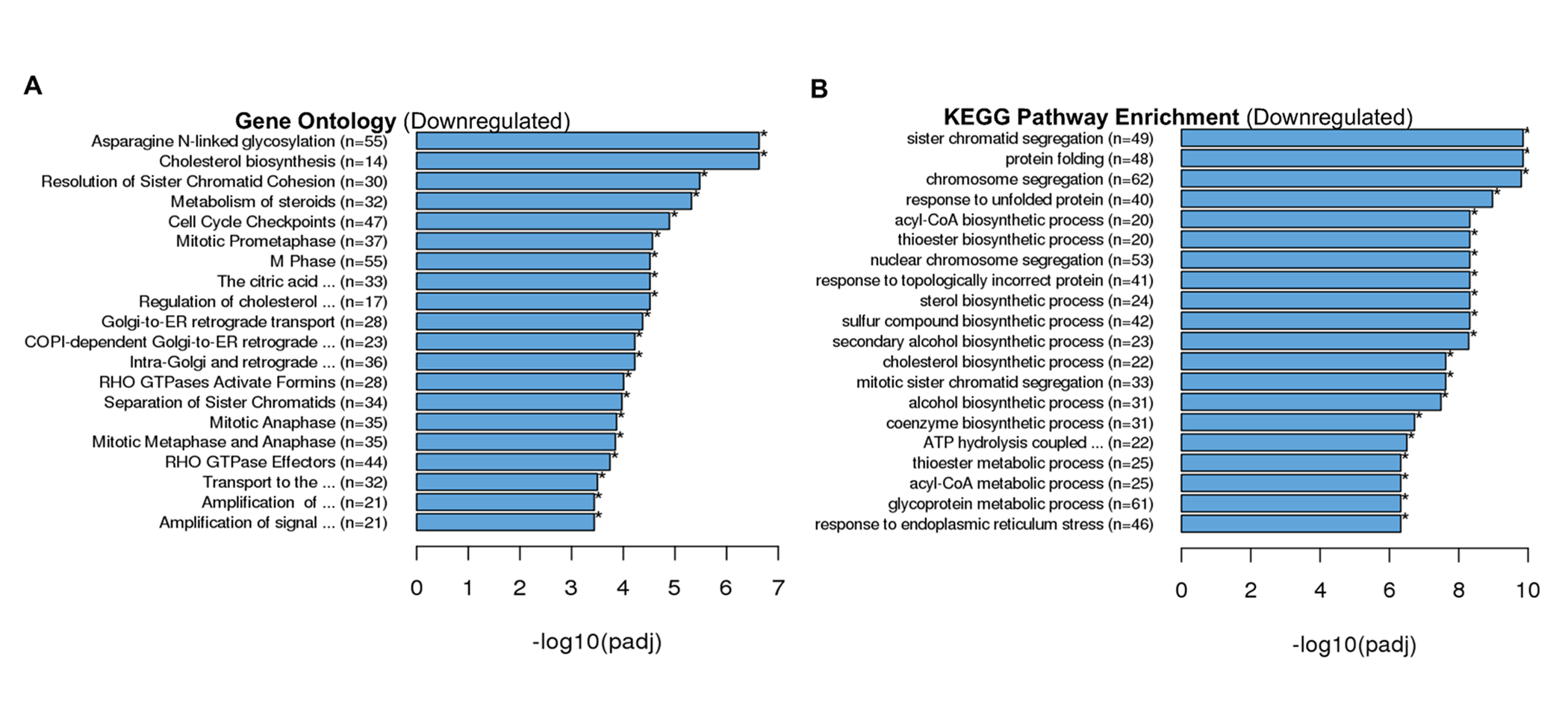

Supplement: S1 Fig — A) Top 20 downregulated Gene Ontology terms of hPDL cells challenged for 2-hours followed by a 22-hour incubation using the Reactome nomenclature. Statistical significance was assessed using a Kolmogorov-Smirnov test followed by Benjamini-Hochberg correction (p<0.05). B) Top 20 downregulated signaling pathways of hPDL cells challenged for 2-hours followed by a 22-hour incubation using the Kyoto Encyclopedia of Genes and Genomes (KEGG) database. Statistical significance was assessed using a Kolmogorov-Smirnov test followed Benjamini-Hochberg correction (p<0.05). (TIF) [file ppat.1009311.s001.tif]

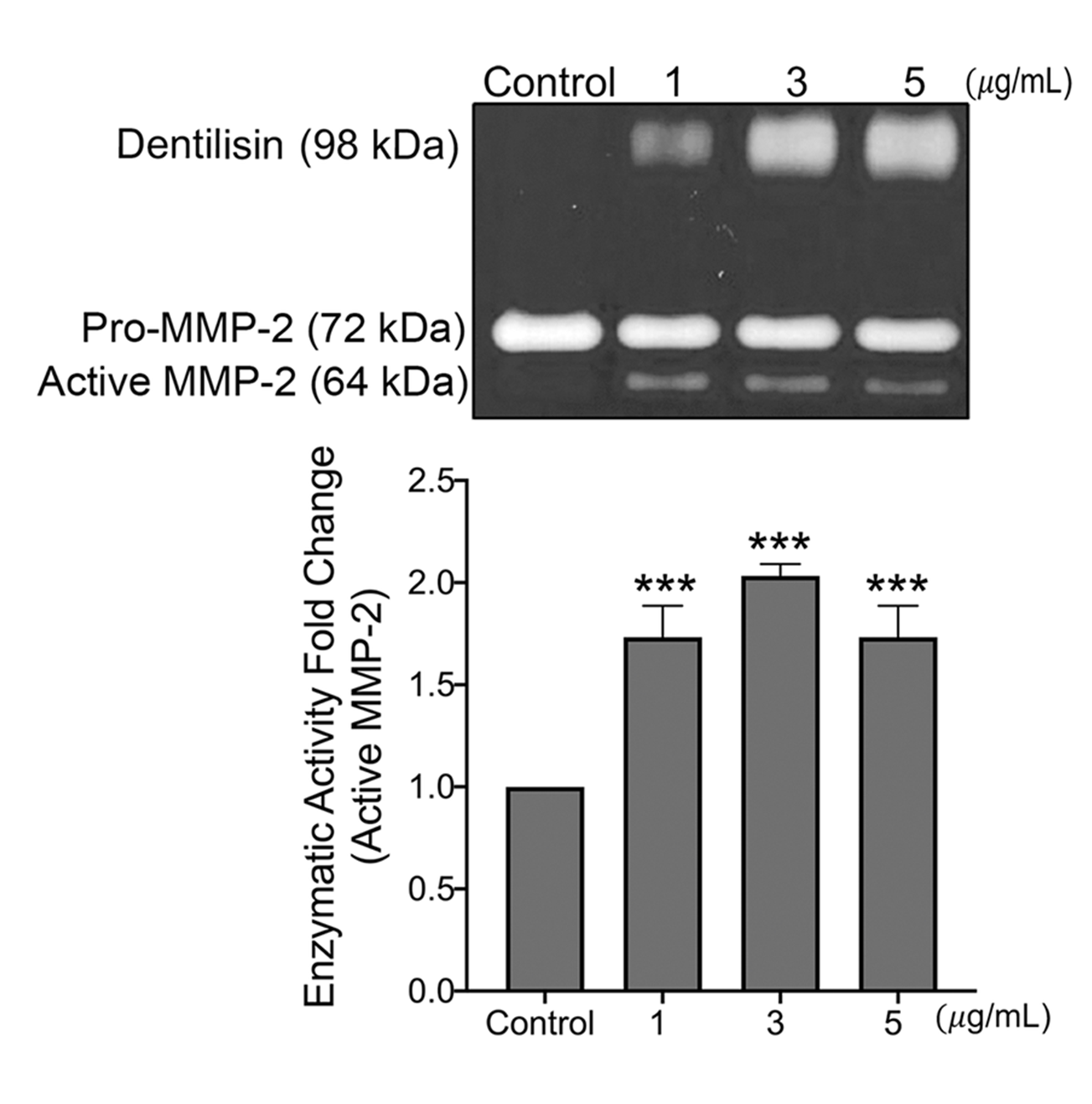

Supplement: S2 Fig — Healthy hPDL cells were challenged with purified dentilisin at increasing concentrations (1, 3 and 5 μg/mL) for 2-hours followed by a 22-hour incubation in MEM-α media free of FBS and supplemented with 1% P/S. Conditioned media from these cells were used to assess the enzymatic activity of Active MMP-2 (64-kDa) and Dentilisin (98 kDa) using gelatin zymography followed by densitometry analysis using Fiji. Statistical significance was determined using a One-Way ANOVA. Bars represent ± SD of mean values (n = 3). ***p < .001 versus control. (TIF) [file ppat.1009311.s002.tif]

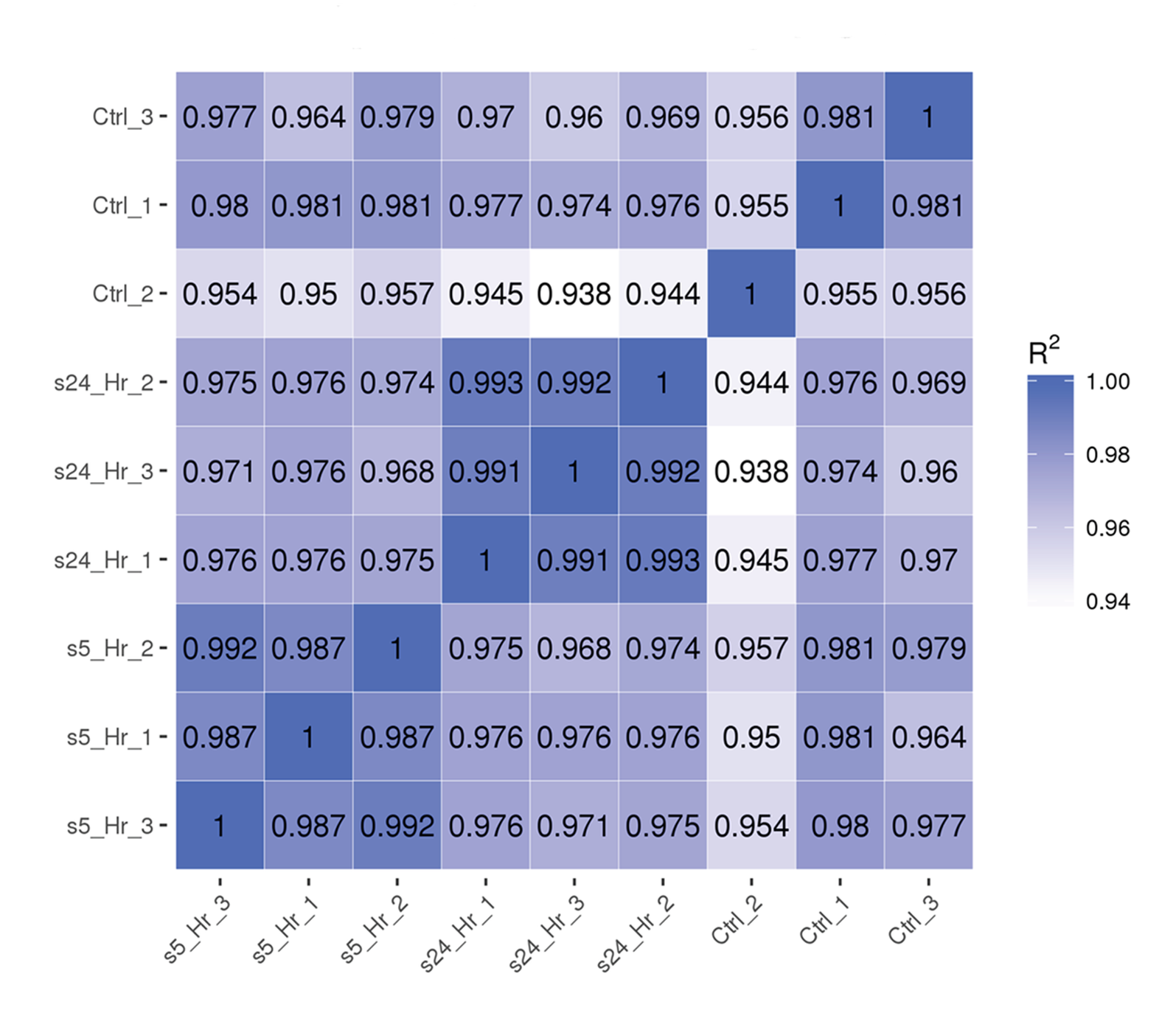

Supplement: S3 Fig — The figure displays a Pearson’s plot visualizing the correlation between samples. Scale bar represents the range of the correlation coefficients (R) displayed. (TIF) [file ppat.1009311.s003.tif]
